# Determination of Caffeine in Energy Drinks Using a Composite Modified Sensor Based on Magnetic Nanoparticles

**DOI:** 10.3390/molecules30102219

**Published:** 2025-05-20

**Authors:** Katarzyna Tyszczuk-Rotko, Aleksandra Liwak, Aleksy Keller

**Affiliations:** Institute of Chemical Sciences, Faculty of Chemistry, Maria Curie-Skłodowska University in Lublin, 20-031 Lublin, Poland; liwakaleksandra@gmail.com (A.L.); alekskeller@op.pl (A.K.)

**Keywords:** caffeine analysis, boron-doped diamond electrode modified with nanocomposite based on Fe_3_O_4_ magnetic nanoparticles in Nafion and electrochemically modified with bismuth film, differential-pulse adsorptive stripping voltammetry, energy drinks

## Abstract

A new voltammetric sensor (BDDE/Nafion@Fe_3_O_4_/BiF) was fabricated by applying a nanocomposite drop of Fe_3_O_4_ magnetic nanoparticles in Nafion onto the polished boron-doped diamond electrode (BDDE) surface. Then, after drying (5 min at room temperature), the electrode was electrochemically modified with bismuth film (BiF) during in situ analysis. The Nafion@Fe_3_O_4_/BiF modification of the BDDE contributes to the acquisition of the highest differential-pulse adsorptive stripping voltammetric (DPAdSV) signals of caffeine (CAF) due to the improvement of electron transfer and the increase in the number of active sites on which CAF can be adsorbed. The DPAdSV signals exhibited a linearly varied oxidation peak with the CAF concentration range between 0.5 and 10,000 nM, leading to the 0.043 and 0.14 nM detection and quantification limits, respectively. The practical applicability of the DPAdSV procedure using the BDDE/Nafion@Fe_3_O_4_/BiF was positively confirmed with commercially available energy drinks.

## 1. Introduction

Caffeine (3,7-dihydro-1,3,7-trimethyl-1H-purine-2,6-dione, CAF) is an alkaloid that belongs to *N*-methyl derivatives of xanthine. It occurs naturally and can be found in many plant products, such as coffee and cacao beans, tea and yerba mate leaves, cola nuts, and guarana berries. It is also known as theine, mateine, and guaranine, depending on where it originates from. CAF is one of the most widely used chemical compounds in the food industry, mainly as an additive in beverages such as cola and energy drinks. It can also be found in many drugs, often combined with acetaminophen. Such drugs are especially used to treat pain coming from various sources, e.g., headaches (also migraines), muscle pain, and toothaches. In addition to treating pain, caffeine is used to treat asthma and nasal congestion as well. It is also found in supplements that support weight loss [1,2,3,4]. Due to its very wide distribution in food and medical products, CAF is believed to be the most commonly consumed drug in everyday life worldwide [5].

CAF does not have any nutritional value, but it shows a particular effect on the human body. It mainly affects the central nervous system and cardiovascular system. In moderate doses, it supports concentration, observation skills, and memory. It reduces the feeling of drowsiness and fatigue and can be used to restore consciousness in states of fainting. CAF consumption may also cause mood improvement. Its effect on the cardiovascular system shows itself through narrowing the blood vessels and increasing the blood pressure. It reduces inflammatory reactions in the body, improves glucose metabolism, and increases gastric acid secretion. However, larger doses of CAF have a harmful effect on human health. It can cause hyperactivity, insomnia, nausea and vomiting, convulsions, heart diseases, depression, and even mutations. An overdose of CAF may be fatal. A lethal dose is estimated to be 170 mg/kg body weight [3,6,7].

As mentioned above, CAF is widely used as an additive in many beverages, mainly energy drinks. Those drinks are very often readily consumed by many young adults, adolescents, and even children, who are most at risk of the side effects of CAF overdose. Consumption of energy drinks has been reported in connection with many harmful effects, e.g., diabetes, sleeplessness, seizures, cardiac, and mood disorders. Moreover, the cessation of CAF intake may lead to withdrawal symptoms, such as headache, lower concentration, fatigue, muscle stiffness, nausea, irritability, and depression. The intensity of those symptoms may be severe. Due to the harmful effects of CAF overconsumption on human health, the advertisement and sales of energy drinks have been restricted in some countries [8,9,10]. For example, in Poland, from 1 January 2024, the sale of energy drinks to children and adolescents under 18 is prohibited. The relevant Act defines it as a beverage that is a food, included in the Polish Classification of Products and Services, which “contains caffeine in a proportion exceeding 150 mg/L or taurine, excluding substances occurring naturally in them” [11].

In conclusion, it is undeniable that CAF has a tremendous impact on the human body. If we add to this its common occurrence in our life and the legal norms, it becomes very important to develop fast, simple, sensitive, and accurate methods for the determination of this compound in various samples, including food and beverages. So far, many different methods have been developed, e.g., using high performance liquid chromatography (HPLC) [12], thermal desorption–gas chromatography mass spectrometry (TD-GC/MS) [13], and voltammetry [1,2,3,4,5,6,7]. Due to the much less expensive equipment, the voltammetric methods are cheaper compared to others, which is a great advantage. Voltammetric measurements are also characterized by higher sensitivity and accuracy as well as lower detection and quantification limits (LODs and LOQs, respectively). Moreover, using screen-printed electrodes (SPEs), it is possible to develop and use voltammetric methods that are suitable for field analysis [7]. Many different approaches have been taken in voltammetric methods of CAF analysis. Nowadays, electrode surface modifications are used very often. These modifications can be realized in many ways, such as deposition of a metal film (e.g., the frequently used bismuth film, BiF) [7], deposition of metallic nanoparticles (e.g., palladium nanoparticles, PdNPs) [14], and coating the surface with a cation exchanger (e.g., Nafion) [5] or carbon nanomaterials (e.g., single-walled carbon nanotubes, SWCNTs) [6]. Such modifications can be combined in many ways to achieve even better results [1,15,16]. The comparison of some methods used for CAF analysis is presented in Table 1.

Recently, magnetic nanoparticles (MNPs) have become increasingly popular in electrochemical analysis. Due to their many advantages, Fe_3_O_4_ MNPs are one of the most widely used magnetic nanomaterials to modify the electrode surface. They are characterized by high surface area, biocompatibility, easy preparation, high absorption ability, and low toxicity. Moreover, they promote a very fast electron transfer between the sensor and the active site of the redox reaction [17,18].

In this paper, we propose a new composite based on magnetic nanoparticles and Nafion to modify the surface of a boron-doped diamond electrode. The electrode was further modified with electrochemically deposited bismuth film (BDDE/Nafion@Fe_3_O_4_/BiF). This way, we acquired a sensitive sensor, which is easy to prepare. Our study aimed to test the proposed electrode modification in the determination of CAF in energy drinks. During our studies, we performed many measurements using differential-pulse adsorptive stripping voltammetry (DPAdSV) to optimize the procedure. It was shown that the use of the BDDE/Nafion@Fe_3_O_4_/BiF increases the oxidation peak currents of CAF. Moreover, we obtained a very low limit of detection (LOD) and limit of quantification (LOQ). The analytical performance of the proposed sensor was tested in the determination of CAF in energy drink samples. The analysis was fast, and the results were found to be satisfactory.

## 2. Results and Discussion

### 2.1. Comparison of the Electrodes

**Figure 1:**
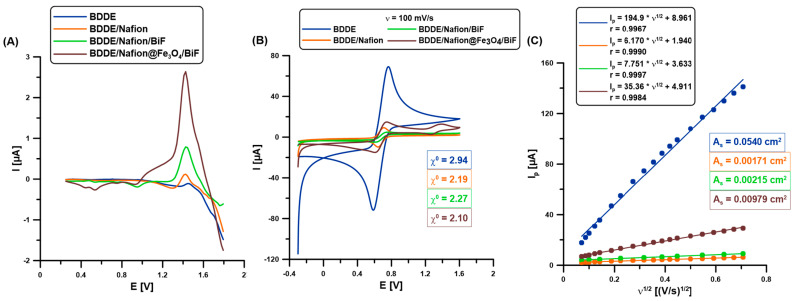
(**A**) The DPAdSVs of 0.1 µM CAF on the BDDE (blue curve), BDDE/Nafion (red curve), BDDE/Nafion/BiF (green curve), and BDDE/Nafion@Fe_3_O_4_/BiF (brown) in 0.1 M H_2_SO_4_ (0 or 6.0 µM Bi(III), E_dep.Bi+acc.CAF_ of −0.95 V, t_dep.Bi+acc.CAF_ of 60 s, ΔE_A_ of 75 mV, ʋ of 175 mV/s, and t_m_ of 4 ms. (**B**) CVs (ʋ = 100 mV/s) in 0.1 M KCl and 5.0 mM [Fe(CN)_6_]^3−/4−^ for all the tested electrodes. (**C**) The relationship between the anode peak (I_p_) and the square root of ʋ (ʋ^1/2^) was determined for the examined sensors (ʋ of 5–500 mV/s).

In preliminary studies, the analytical signals of caffeine (CAF, 0.1 µM) on an unmodified boron-doped diamond electrode (BDDE), a BDDE modified with a Nafion layer (BDDE/Nafion) and an in situ deposited bismuth film (BDDE/Nafion/BiF), and a BDDE modified with a nanocomposite consisting of magnetic nanoparticles (Fe_3_O_4_) and Nafion, and electrochemically modified with bismuth film (BDDE/Nafion@Fe_3_O_4_/BiF) (Figure 1A,B), were compared. The choice of the BDDE, Nafion, and Bi film modification was not accidental since it was based on the results obtained in our previous study [1]. In that paper, among others, it was demonstrated that: (1) the use of BDDE instead of a glassy carbon electrode provided a lower background current as well as a better repeatability of the paracetamol signal, (2) the advantages of using the Nafion film was connected with preconcentration of caffeine in the polymer layer, (3) higher and better shaped signals of paracetamol and caffeine were obtained when the BDDE/Nafion was modified with BiF instead the PbF. The differential-pulse adsorptive stripping voltammograms (DPAdSVs, Figure 1A) show the presence of a CAF peak at a potential of about 1.5 V on all the tested electrodes. The results obtained in our previous study were confirmed; the Nafion layer causes an increase in the analytical signal of CAF due to its preconcentration in the polymer layer (BDDE vs. BDDE/Nafion, 0.089 vs. 0.29 µA) [1,19]. Furthermore, the bismuth film modification contributed to another CAF signal increase (BDDE/Nafion vs. BDDE/Nafion/BiF, 0.29 vs. 0.77 µA). In the previous work, we did not explain what this is related to [1]. This work explained it by using cyclic voltammetric studies (the results described below). Additionally, we observed a significant increase in the CAF signal (BDDE/Nafion/BiF vs. BDDE/Nafion@F_3_O_4_/BiF, 0.77 vs. 1.96 µA) after introducing the nanocomposite based on commercially available Fe_3_O_4_ nanoparticles onto the electrode surface. This is most likely related to the excellent adsorption and catalytic properties of Fe_3_O_4_ nanoparticles [20,21].

The changes in the electrochemical properties of all the studied electrodes were also examined using cyclic voltammetry (CV, solution: 0.1 M KCl and 0.5 mM [Fe(CN)_6_]^3−/4−^, at a scan rate (ʋ) of 5–500 mV/s). Figure 1B demonstrates the enhancement of the analytical signals of Fe(II) and Fe(III) with each subsequent component of the electrode surface modifier (the red, green, and brown curves, respectively). However, the highest peak intensities of Fe(II) and Fe(III) were on the unmodified BDDE, which results in the largest active surface area (A_s_, Figure 1C) [22]. Nonetheless, this does not translate into the highest CAF signal on the BDDE due to the weakest electron transfer between the electrode surface and solution (the highest relative separation of the oxidation and reduction iron peaks, χ^0^ of 2.94). Based on the determined A_s_ and χ^0^ values for all tested electrodes (Figure 1B,C), it can be stated that among the BDDE surface modifiers used, Nafion@Fe_3_O_4_/BiF modification contributes to the acquisition of the highest DPAdSV signals of CAF due to the improvement of electron transfer (the lowest χ^0^ value of 2.10) and the increase in the number of active sites on which CAF can be adsorbed (the highest A_s_ value among the modified electrodes—BDDE/Nafion, BDDE/Nafion/BiF and BDDE/Nafion@Fe_3_O_4_/BiF).

### 2.2. Surface Modifier Composition Optimization

**Figure 2:**
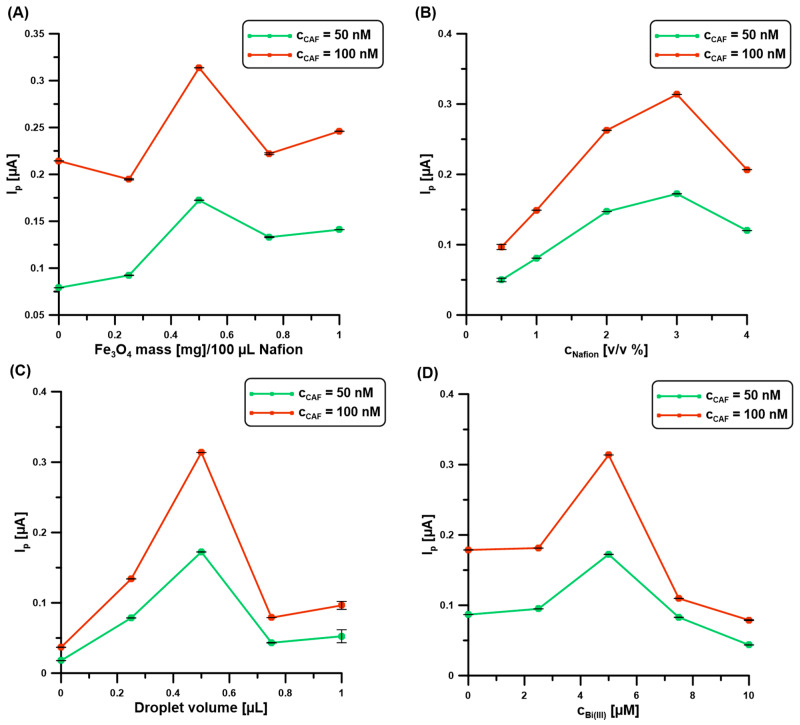
The relationship between the 50 and 100 nM CAF signals, and (**A**) mass of Fe_3_O_4_ in the 100 μL of 3% Nafion, (**B**) concentration of Nafion, (**C**) droplet volume, and (**D**) concentration of Bi(III) in the supporting electrolyte (0.1 M H_2_SO_4_). DPAdSV parameters: E_dep.Bi+acc.CAF_ of −0.95 V, t_dep.Bi+acc.CAF_ of 60 s, ΔE_A_ of 75 mV, ʋ of 175 mV/s, and t_m_ of 4 ms. The SD values were calculated for n = 5.

In the next stage of the research, the composition of the modifier (Nafion@Fe_3_O_4_/BiF) of the BDDE was optimized. The effect of parameters such as mass of Fe_3_O_4_ (Figure 2A) in the 100 μL of 3% Nafion, concentration of Nafion (Figure 2B), droplet volume (Figure 2C), and concentration of Bi(III) in the supporting electrolyte (Figure 2D), on the 50 and 100 nM CAF signals, was examined. As can be seen, with the increase of Fe_3_O_4_ content to 0.5 mg, Nafion concentration to 3%, and Bi(III) to 5 µM, the analytical signals of CAF increase. This is related to the improvement of the adsorption capacity of the modifier. Above these values, a decrease in CAF signals was observed, which is associated with the blocking of the active surface of the electrode. As for the droplet size, the value of 0.5 µL was selected for further measurements. Too small a drop volume causes the entire geometric surface of the BDDE (0.0707 cm^2^) to be uncovered, while too large a drop volume results in the formation of too thick a layer and/or spreading beyond the active surface. The following parameters were selected: 0.5 mg of Fe_3_O_4_ in 100 μL of 3% Nafion, a droplet volume of 0.5 µL, and a Bi(III) concentration of 5 µM.

### 2.3. Type and Concentration of the Base Electrolyte

**Figure 3:**
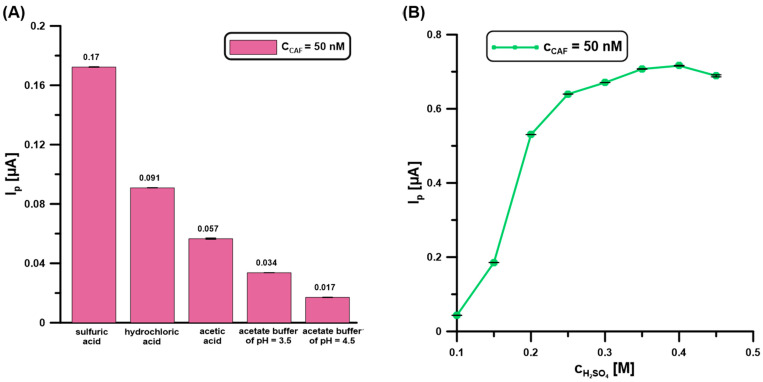
The influence of type (**A**) (0.1 M H_2_SO_4_, HCl, CH_3_COOH, CH_3_COONa/CH_3_COOH of pH = 3.5 and 4.5 0.1), and (**B**) concentration of H_2_SO_4_ on the DPAdSV response of CAF (50 nM). DPAdSV parameters: E_dep.Bi+acc.CAF_ of −0.95 V, t_dep.Bi+acc.CAF_ of 60 s, ΔE_A_ of 75 mV, ʋ of 175 mV/s, and t_m_ of 4 ms. The standard deviation (SD) values were calculated for n = 5.

In the next stage of research, the type (Figure 3A) and concentration (Figure 3B) of the base electrolyte were tested. According to the literature data, the three main advantages are using a low pH during CAF determination: a relatively high analytical signal, relatively little interference from background current (probably oxygen evolution), and freedom from extraneous peaks due to possible adsorption [23]. Therefore, an acidic environment for the CAF (50 nM) analysis at the BDDE/Nafion@Fe_3_O_4_/BiF was studied, including H_2_SO_4_, HCl, CH_3_COOH, and CH_3_COONa/CH_3_COOH of pH = 3.5 and 4.5 (Figure 3A). At this stage of research, a constant electrolyte concentration of 0.1 M was used. The increase in the analytical CAF signal is visible with the decrease in the electrolyte pH value. The highest CAF signal was obtained for 0.1 M H_2_SO_4_. The data described in the literature are also confirmed at the BDDE/Nafion@Fe_3_O_4_/BiF. CAF is a weak electrolyte, and its ionization is highly influenced by the solution’s pH. Based on the CAF pKa values (pKa_1_ = 0.6–0.7 and pKa_2_ = 14), at a low pH a higher concentration of the protonated form is present, which is easily incorporated in the cation-exchange Nafion polymer [24,25]. This contributes to the increase in the CAF analytical signal due to more effective adsorption.

Then, the effect of electrolyte concentration on the analytical signal of CAF (50 nM) was examined. The concentration of H_2_SO_4_ varied from 0.1 to 0.45 M (Figure 3B). The CAF signal increases with increasing electrolyte concentration up to 0.4 M, and then it is almost constant. For further measurements, 0.4 M was selected.

### 2.4. Electrochemical Performance of CAF

The electrochemical performance of CAF on Nafion-modified BDDEs is very well described in the literature—the oxidation mechanism reaction of CAF proceeds by an overall 4e^−^, 4H^+^ process. The first step, the 2e^−^, 2H^+^ oxidation of the C-8 to N-9 bonds to give the substituted uric acid, is followed by the immediate 2e^−^, 2H^+^ oxidation to the 4,5-diol analog of uric acid, which rapidly fragments [23,26,27].

**Figure 4:**
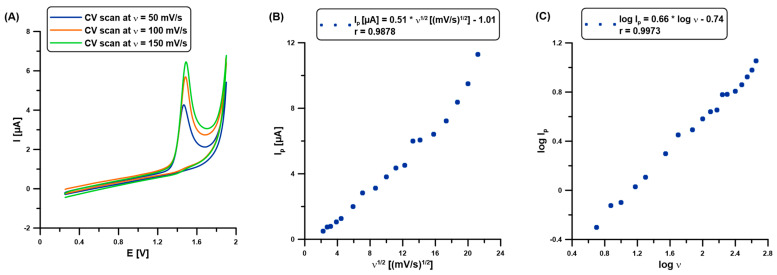
(**A**) CVs at the BDDE/Nafion@Fe_3_O_4_/BiF in 0.1 M H_2_SO_4_ and 100 mM CAF. The relationship between (**B**) I_p_ and ʋ^1/2^, and (**C**) log I_p_ and log ʋ (ʋ: 5–450 mV/s).

Further experiments were performed to study the transport characteristics of CAF in the Nafion@Fe_3_O_4_/BiF layer. Figure 4A shows the selected CVs (ʋ: 50, 100, and 150 mV/s), which are all applied in the scan rate range (ʋ: 5–450 mV/s). The CVs were registered in 0.1 M H_2_SO_4_ and 100 mM CAF at the BDDE/Nafion@Fe_3_O_4_/BiF. The sensor was prepared in a separate solution (0.1 M H_2_SO_4_ containing 5 µM Bi(III), E_dep.Bi_ of −0.95 V, t_dep.Bi_ of 60 s) once before CV measurements. The clear well-formed CAF analytical signals are visible at a potential of approx. 1.4 V. The current response (I_p_) was found to be not directly proportional to the ʋ^1/2^ in a wide range of ʋ between 5 and 450 mV/s (r = 0.9878), which demonstrates that the electrode oxidation reaction of CAF was an adsorption-controlled process at the BDDE/Nafion@Fe_3_O_4_/BiF (Figure 4B). It was confirmed by the slope value (0.66), which was higher than the theoretical one of 0.5 (Figure 4C).

### 2.5. Deposition and Accumulation Step

**Figure 5:**
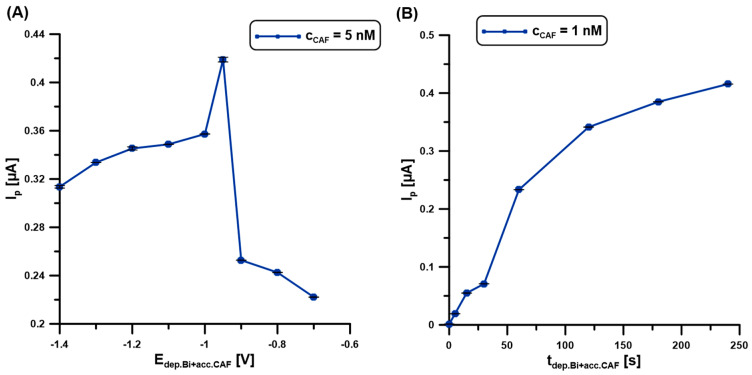
The influence of E_dep.Bi+acc.CAF_ (**A**), and t_dep.Bi+acc.CAF_. (**B**) on the DPAdSV responses of 5 (**A**) and 1 nM (**B**) CAF. DPAdSV parameters: t_dep.Bi+acc.CAF_ of 60 s (**A**), E_dep.Bi+acc.CAF_ of −0.95 V (**B**), ΔE_A_ of 75 mV, ʋ of 175 mV/s, and t_m_ of 4 ms. The SD values were calculated for n = 5.

The BiF deposition and CAF accumulation steps were performed simultaneously to reduce the measurement time. In the first stage of the experiments, the impact of the potential of simultaneous BiF deposition and CAF accumulation (E_dep.Bi+acc.CAF_) on the DPAdSV response was studied (Figure 5A). The changes in the potential range from −0.7 to −1.4 V significantly affect the CAF signal (5 nM). It is worth adding that the time between the application of the potential and the working electrode was equal to 60 s. The highest signal with satisfactory repeatability was obtained at a potential of −0.95 V. Then, the time of simultaneous BiF deposition and CAF accumulation (t_dep.Bi+acc.CAF_) was changed from 0 to 240 s (Figure 5B). In the case of the BDDE/Nafion@Fe_3_O_4_/BiF, the voltammetric peak height of CAF (1 nM) increases with time, which is caused by the increasing amount of Bi particles deposited on the surface and the increased efficiency of molecule accumulation on/in the modifier layer. To shorten the analysis time, a time of 60 s was chosen. However, studies show that the detection limit can be reduced by extending the BiF deposition time and CAF accumulation.

### 2.6. Technique Parameters

**Figure 6:**
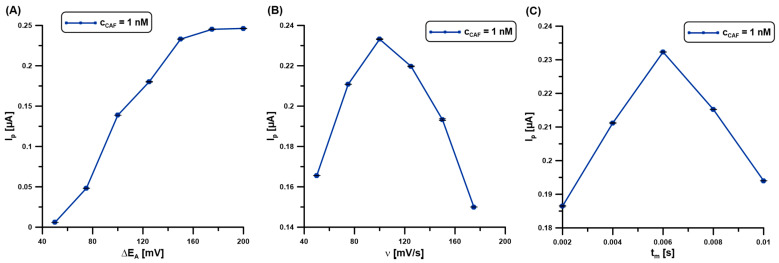
The influence of ΔE_A_ (**A**), ʋ (**B**), and t_m_ (**C**) on the DPAdSV responses of 1 nM CAF. DPAdSV parameters: E_dep.Bi+acc.CAF_ of −0.95 V and t_dep.Bi+acc.CAF_ of 60 s. The SD values were calculated for n = 5.

Due to achieving optimal sensitivity, the following parameters of the DPAdSV technique were optimized: the amplitude (ΔE_A_), the scan rate (υ), and the modulation time (t_m_). The effect of ΔE_A_ (25–200 mV) on the 1 nM CAF signal was evaluated (Figure 6A). The peak current of CAF was observed to increase with increasing ΔE_A_ up to 150 mV, while the signal intensity had similar values. For the selected value of ΔE_A_ (150 mV), ʋ was now varied from 50 to 175 mV/s and the changes in the CAF signal (1 nM) were observed (Figure 6B). With the increase in ν, the CAF signals increased, reaching the highest intensity at ʋ of 100 mV/s. The effect of t_m_ on the DPAdSV responses of 1 nM CAF was evaluated in the range of 2–10 ms. The maximum CAF signals were recorded at 6 ms (Figure 6C).

### 2.7. Repeatability and Reproducibility

**Figure 7:**
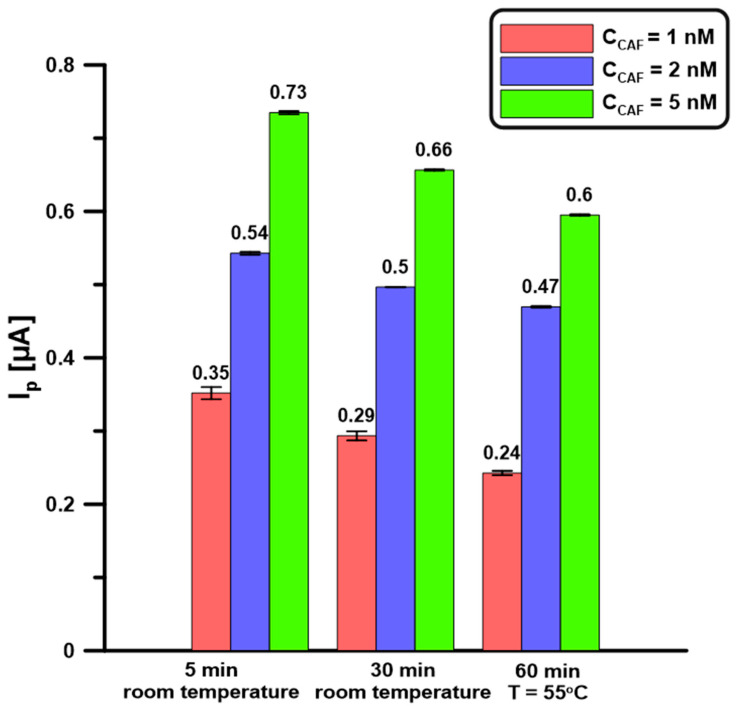
BDDE/Nafion@Fe_3_O_4_ drying procedures. DPAdSV parameters: E_dep.Bi+acc.CAF_ of −0.95 V, t_dep.Bi+acc.CAF_ of 60 s, ΔE_A_ of 150 mV, ʋ of 100 mV/s, and t_m_ of 6 ms. The SD values were calculated for n = 5.

Repeatability was evaluated with successive 1, 2, and 5 nM CAF measurements (n = 5) at the BDDE/Nafion@Fe_3_O_4_/BiF. The electrodes were prepared by applying 0.5 µL of the nanocomposite onto the BDDE surface, then drying it at room temperature for 5 or 30 min, or in a drying oven (T = 55 °C) for 60 min. Next, the electrode was placed in a basic electrolyte containing Bi(III) (5 µM) and the analyte, a BiF film was deposited, and CAF was determined. There was no significant improvement in signal repeatability or intensity with increasing the time or temperature of drying the Nafion@Fe_3_O_4_ layer on the BDDE surface (Figure 7). To shorten the electrode preparation time, drying for 5 min at room temperature was chosen. The obtained RSD values in these conditions for 1, 2, and 5 nM CAF are 2.4, 2.0, and 1.3%, respectively, which confirms the satisfactory signal repeatability.

Electrode-to-electrode reproducibility was calculated for three independently prepared BDDE/Nafion@Fe_3_O_4_/BiF based on the 5 nM CAF signal intensities (n = 15). The RSD value of 5.9% confirms satisfactory reproducibility.

### 2.8. Sensitivity, Selectivity and Application

**Figure 8:**
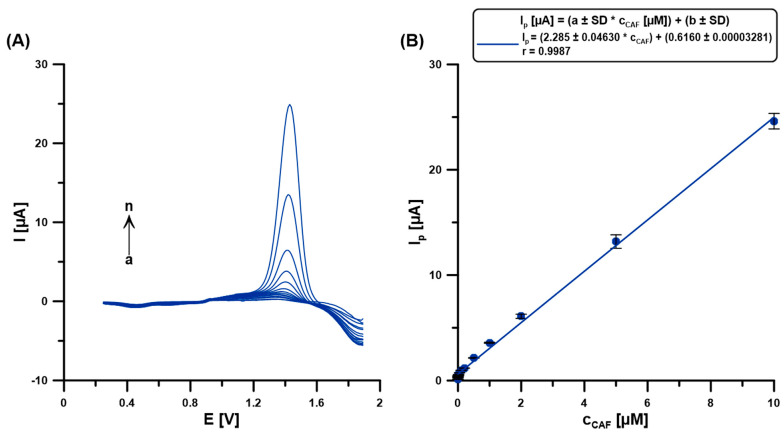
(**A**) The DPAdSVs registered at the BDDE/Nafion@Fe_3_O_4_ in 0.1 M H_2_SO_4_ containing 5 µM Bi(III) and CAF (a → n, 0.0005–10 µM). (**B**) The calibration curve. DPAdSV parameters: E_dep.Bi+acc.CAF_ of −0.95 V, t_dep.Bi+acc.CAF_ of 60 s, ΔE_A_ of 150 mV, ʋ of 100 mV/s, and t_m_ of 6 ms. The SD values were calculated for n = 5.

Measurements for the calibration curve were made in the base electrolyte solution (0.1 M H_2_SO_4_) containing 5 µM Bi(III) after introducing a CAF standard solution of known concentration. The obtained calibration curve is characterized by a very wide linear range from 0.5 to 10,000 nM (0.0005–10 µM) CAF (Figure 8). The equations, LOD = 3 SD_a_/b and LOQ = 10 SD_a_/b (SD_a_—standard deviation of intercept (n = 3), b—slope of the linear regression equation), were used to determine the limits of detection (LOD = 0.043 nM) and quantification (LOQ = 0.14 nM) of CAF calculation. Table 1 compares the selected methods for CAF determination [1,3,6,12,13]. The results collected in this table confirm that the developed procedure at the BDDE/Nafion@Fe_3_O_4_/BiF offers a wide linear range of the calibration curve and one of the lowest LODs.

**Figure 9:**
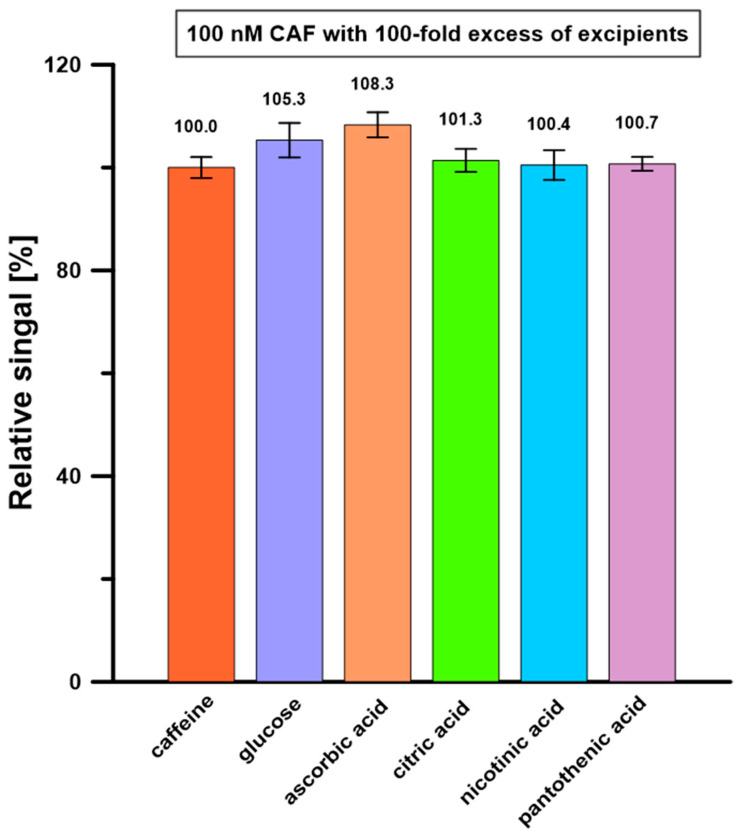
Influence of 100-fold excess of possible interferents on 100 nM CAF signals. The SD values were calculated for n = 5.

Possible interferents were selected for the selectivity study based on the composition presented on the labels of the analyzed energy drinks. The influence of glucose, vitamin C, vitamin B3 (nicotinic acid), vitamin B5 (pantothenic acid), and citric acid on the 50 nM CAF signals was examined. It was found that a 100-fold excess of these substances in relation to the CAF concentration did not cause a change in its signal by more than ±10% (Figure 9). Moreover, none of the possible interferents gave an oxidation signal in the recorded potential range. The results suggested that the developed procedure at the BDDE/Nafion@Fe_3_O_4_/BiF can accurately determine CAF in energy drink samples.

The developed procedure at the BDDE/Nafion@Fe_3_O_4_/BiF was optimized for its application in determining CAF in energy drink samples. The samples were analyzed by the standard addition method. The samples were only degassed and diluted in distilled water at a ratio of 1:10 before being introduced into the electrochemical cell. Sample 1 with a CAF content of 32 mg/100 mL (1.65 mM), as declared by the manufacturer, was introduced in 5 µL, and sample 2 with the content of 14 mg/100 mL (0.72 mM), as declared by the manufacturer, was introduced in 10 µL. The results (Table 2) show a satisfactory degree of precision (coefficients of variation: 7.5 and 1.8%) and accuracy (recoveries: 97.0 and 100.0%) of the developed DPAdSV procedure.

## 3. Materials and Methods

### 3.1. Instrumentations

For the voltammetric studies, an electrochemical analyzer µAutolab (Eco Chemie, Utrecht, The Netherlands) was used. A commercially available BDD electrode (Windsor Scientific Ltd., Slough, Berkshire, UK) was purchased in an inert polytetrafluoroethylene (PTFE, Teflon) body with an inner diameter of 3 mm (D-086-SA, boron doping level of 1000 ppm, electrical resistivity of 0.075 Ωm). Before applying onto the BDDE surface nanocomposite layer, it was polished using 0.3 µm alumina slurry on a Buehler polishing pad (Lake Bluff, IL, USA). The electrochemical cell also consisted of a silver chloride electrode (3 M KCl, reference) and a Pt wire (auxiliary electrode).

### 3.2. Reagents

The caffeine (CAF) solutions were prepared by dissolving the reagent obtained from Merck (Darmstadt, Germany) in deionized water or by diluting solutions of higher concentration and storing them at 4 °C in the dark until used. Fe_3_O_4_ nanoparticles (nanopowder, 50–100 nm particle size) were obtained from Sigma-Aldrich (Darmstadt, Germany). The 0.1 M H_2_SO_4_ and 1 mM Bi(III) solutions were prepared from reagents purchased from Merck (Darmstadt, Germany). Nafion (etrafluoroethylene-perfluoro-3,6-dioxa-4-methyl-7-octenesulfonic acid copolymer, 5% *w*/*v* solution) was obtained by diluting a reagent purchased from Aldrich (Darmstadt, Germany) with ethanol (POCH, Lublin, Poland) to reach a 3% concentration. The stock standard solution of glucose, vitamin C, vitamin B3, vitamin B5, and citric acid (Sigma-Aldrich, Darmstadt, Germany) was prepared in ultra-purified water (>18 MΩ cm, Milli-Q system, Millipore, Gillingham, UK).

### 3.3. BDDE/Nafion@Fe_3_O_4_/BiF Preparation and CAF Analysis

In the first stage, a nanocomposite containing 0.5 mg Fe_3_O_4_ in 100 µL of 3% (*v*/*v*) Nafion (in ethanol) was prepared. It was placed in an ultrasonic bath for 2 h. Then, 0.5 µL of such a nanocomposite was applied with an automatic pipette onto the polished and dried BDDE surface. Next, after 5 min at room temperature, the electrode was placed in a solution containing 0.1 M H_2_SO_4_, 5 µM Bi(III), and CAF. The bismuth film was deposited, and CAF was accumulated simultaneously at a potential of −0.95 V (E_dep.Bi+acc.CAF_) for 60 s (t_dep.Bi+acc.CAF_) under string. The differential-pulse adsorptive stripping voltammograms were registered in the range of 0.25 to 1.85 V with the amplitude (ΔE_A_) of 150 mV, the scan rate (υ) of 100 mV/s, and the modulation time (t_m_) of 6 ms. The background was subtracted from each measurement.

The BDDE/Nafion@Fe_3_O_4_ was prepared before each series of measurements, i.e., a given stage of procedure optimization, measurements to the calibration curve, and sample analysis. The bismuth film was applied in situ, during the CAF determination.

## 4. Conclusions

The voltammetric sensor (BDDE/Nafion@Fe_3_O_4_/BiF) based on Fe_3_O_4_ nanoparticles in the cation-exchange Nafion polymer and electrochemically deposited bismuth film was fabricated for the sensitive and selective determination of caffeine (CAF). It was found that the Nafion@Fe_3_O_4_/BiF modification contributes to the acquisition of the highest differential-pulse adsorptive stripping voltammetric (DPAdSV) signals of CAF due to the improvement of electron transfer (the lowest χ^0^ value) and the increase in the number of active sites on which CAF can be adsorbed (the highest A_s_ value among the modified electrodes—BDDE/Nafion, BDDE/Nafion/BiF and BDDE/Nafion@Fe_3_O_4_/BiF). The developed DPAdSV procedure at the BDDE/Nafion@Fe_3_O_4_/BiF offers a wide linear range of the calibration curve and one of the lowest limits of detection. The analytical performances of the BDDE/Nafion@Fe_3_O_4_/BiF were satisfactory as evidenced in the repeatability, reproducibility, and selectivity. The practical applicability of the DPAdSV procedure using the BDDE/Nafion@Fe_3_O_4_/BiF was positively confirmed with commercially available energy drinks.

## Figures and Tables

**Table 1:** Comparison of the methods used for the CAF analysis in various samples.

Method (Sensor)	Linear Range [nM]	LOD [nM]	Sample	Ref.
RP-HPLC with UV detection	51,500–412,000	875	Tablets	[12]
TD-GC/MS	51,500–2,570,000	2350	Energy drinks, cola	[13]
DPV (Bi/Nafion/BDDE)	10–20,000	1.14	Energy drinks, cola, tea, coffee	[1]
DPAdSV (SPCE/CNFs)	200–1000	56	Energy drink, cola	[3]
DPV (SWCNT/CCE)	250–100,000	120	Mineral water	[6]
DPAdSV (BDDE/Nafion@Fe_3_O_4_/BiF)	0.5–10,000	0.0431	Energy drinks	This work

RP-HPLC—Reversed-Phase High-Performance Liquid Chromatography; TD-GC/MS—Thermal Desorption–Gas Chromatography Mass Spectrometry; DPV—Differential-Pulse Voltammetry; Bi/Nafion/BDDE—Bismuth particles Nafion covered Boron-Doped Diamond Electrode; DPAdSV—Differential-Pulse Adsorptive Stripping Voltammetry; SPCE/CNFs—Screen-Printed Carbon Electrode coated with Carbon Nanofibers; SWCNT/CCE—Carbon Ceramic Electrode modified with Single-Walled Carbon Nanotubes; BDDE/Nafion@Fe_3_O_4_/BiF—Boron-Doped Diamond Electrode modified with Nafion and Fe_3_O_4_ nanocomposite and Bismuth Film.

**Table 2:** The results of CAF determination in energy drink samples.

CAF Concentration [mM] ± SD (n = 3)			
Sample (Content)	Found DPV	Coefficient of Variation * [%]	Recovery ** [%]
1 (1.65)	1.60 ± 0.12	7.5	97.0
2 (0.72)	0.72 ± 0.013	1.8	100.0

* Coefficient of variation [%] = (SD × 100)/Found DPAdSV, ** Recovery [%] = (Found DPAdSV × 100)/Content specified by the manufacturer.

## Data Availability

The original contributions presented in this study are included in the article. Further inquiries can be directed to the corresponding author.
